# ZFP91: A Noncanonical NF-*κ*B Signaling Pathway Regulator with Oncogenic Properties Is Overexpressed in Prostate Cancer

**DOI:** 10.1155/2016/6963582

**Published:** 2016-11-16

**Authors:** Lukasz Paschke, Karol Jopek, Marta Szyszka, Marianna Tyczewska, Agnieszka Ziolkowska, Marcin Rucinski, Ludwik K. Malendowicz

**Affiliations:** Department of Histology and Embryology, Poznan University of Medical Sciences, 6 Swiecicki St., 60-781 Poznan, Poland

## Abstract

Novel molecular targets are being searched to aid in prostate cancer diagnosis and therapy. Recently, ZFP91 zinc finger protein has been found to be upregulated in prostate cancer cell lines. It is a potentially important oncogenic protein; however only limited data regarding its biological function and expression patterns are available. To date, ZFP91 has been shown to be a key factor in activation of noncanonical NF-*κ*B signaling pathway as well as to be involved in HIF-1*α* signaling in cancer cells. The present study aimed to characterize* ZFP91* expression in prostate cancer specimens. Furthermore, since our earlier reports showed discrepancies between* ZFP91* mRNA and protein levels, we studied this interrelationship in LNCaP and PC-3 prostate cancer cell lines using siRNA mediated knockdown. QPCR analysis revealed marked upregulation of ZFP91 mRNA in the majority of prostate cancer specimens. Transfection of prostate cancer cells with* ZFP91* siRNA resulted in a 10-fold decrease in mRNA levels. On a protein level, however, no inhibitory effect was observed over the time of the cell culture. We conclude that* ZFP91 *is overexpressed in prostate cancer and that potential accumulation of the ZFP91 protein in studied cells may be of importance in prostate cancer biology.

## 1. Introduction

Prostate cancer is worldwide the second most common cancer in men with over million of new cases diagnosed every year and is the fifth leading cause of death of cancer in men (WHO International Agency for Research on Cancer, http://www.iarc.fr/). With the advent of whole genome expression analyses and progress in molecular biology techniques several molecular pathways involved in prostate cancer pathogenesis have been discovered. Still novel targets are being searched to aid in diagnosis and treatment of this complex and heterogeneous disease [1]. In 2014 our team described changes in expression of little-studied ZFP91 zinc finger protein gene (*ZFP91*) in benign prostate hyperplasia (BPH) and in prostate cancer cell lines [2]. Recently found oncogenic properties of this gene prompted us to further study its significance in prostate cancer pathogenesis.

*ZFP91 *gene was discovered in 1995 by Saotome et al. [3]. In 2003, using cDNA microarray screening, Unoki et al. found its overexpression in mononuclear cells from patients with acute myelogenous leukemia (AML) and in several neoplastic blood cell lines [4]. Subsequently, in 2006 an interaction of ZFP91 with ARF tumor suppressor protein (cyclin-dependent kinase inhibitor 2A, isoform 4) was discovered. This protein serves important antioncogenic functions based on p53-dependent cell death or cell cycle arrest in response to oncogenes activation [5]. To date the most important findings regarding* ZFP91* gene functions come from works of Lee and Jin et al. This team patented concepts of ZFP91-based therapies and published a series of papers providing valuable insight into* ZFP91* role in human biology and cancer pathogenesis [6–9].

*ZFP91* expression is positively regulated by agonists of the nuclear factor kappa B (NF-*κ*B) signaling pathway through a promotor sequence located in* ZFP91* gene's 5′ upstream region. On the other hand,* ZFP91* overexpression results in increased NF-*κ*B activity in a dose dependent manner. This effect is also dependent on presence of MAP3K14 protein, known also as NIK (NF-*κ*B-inducing kinase). This is a key kinase in activation of noncanonical (alternative) NF-*κ*B signaling pathway [7, 10]. It has been shown that ZFP91 protein functions as an atypical E3 ubiquitin-protein ligase. Ubiquitination of NIK by ZFP91 leads to its stabilization and activation of the noncanonical NF-*κ*B pathway and this pathway's target genes expression [7, 8]. What is important, available data imply oncogenic activity of the NIK and its overexpression has been associated with cancer pathogenesis in, for example, melanoma, pancreatic, breast, and lung cancer [11].

Oncogenic properties of* ZFP91* gene are not only limited to NIK stabilization and NF-*κ*B signaling pathway activation. Most recently, it has been shown that* ZFP91* is overexpressed in human colon cancer and promotes this cancer progression. Through interaction with NF-*κ*B/p65, ZFP91 protein upregulates hypoxia inducible factor-1*α* (HIF-1*α*). HIF-1*α* is a subunit of a key transcription factor responsible for cellular response to hypoxia and implicated on many levels in cancer pathogenesis and biology [12, 13]. With regard to prostate cancer, HIF-1*α* is overexpressed in actively growing prostate tissues: BPH and prostate cancer [14]. Under hypoxic conditions HIF-1*α* dependent signaling promotes epithelial to mesenchymal transition (EMT) in prostate cancer cells which is proven to play a role in cancer progression and invasiveness [15, 16].

In the current study, the* ZFP91* gene expression was examined in prostate cancer specimens and found to be markedly upregulated. To study further* ZFP91* expression in prostate cancer cells, LNCaP and PC-3 prostate cancer cell lines were transfected with* ZFP91* targeting siRNA. In the result a significant discrepancy between* ZFP91* mRNA level changes and protein levels in these cells was observed. This indicates that ZFP91 protein may be stabilized and accumulated in prostate cancer cells and this effect may be connected with oncogenic properties of* ZFP91*.

## 2. Materials and Methods

### 2.1. Prostate Cancer cDNA Samples

Prostate Cancer cDNA Array III (Origene, HPRT103) was utilized providing samples from 48 prostate cancer patients. Description of every sample includes relevant clinical information, full pathology report, and RNA quality data (data available at http://www.origene.com/qPCR/Tissue-qPCR-Arrays.aspx). In 9 cases tissue samples came from parts of prostate without pathological changes and in remaining 39 cases from parts of prostate with cancer. Each sample was evaluated for mRNA expression of* ZFP91* and of three reference genes: tubulin alpha 1b (*TUBA1B*), 5′-aminolevulinate synthase 1 (*ALAS1*), and *β*2-microglobulin (*B2M*). Out of these three,* TUBA1B* and* ALAS1* were selected using geNorm method as a reference to normalize data. Of note, selected genes were proven to be among the most stable and useful ones for normalization purposes in gene profiling studies of prostate tissues, both malignant and not [17].

### 2.2. Prostate Cancer Cell Lines

Prostate cancer cell lines, LNCaP and PC-3, were obtained from American Type Culture Collection (ATCC, Manassas, USA) and maintained in RPMI-1640 Medium (LNCaP) or F12K Medium (PC-3). Media were purchased from ATCC and supplemented with 10% fetal bovine serum. The cells were grown in 75 cm^2^ flasks at 37°C in a humidified atmosphere of 5% CO_2_. The culture medium was changed every 2 days. When cells reached approximately 80% confluence, they were either subcultured or harvested by 0.25% trypsin-EDTA. Harvested cells were frozen in −80°C for further analyses.

### 2.3. Transfection

LNCaP and PC-3 cells transfection conditions were optimized using siGLO Green Transfection Indicator (Dharmacon, GE Healthcare, Lafayette, USA) and Fluoview FV10i-LIV confocal microscope (Olympus, Melville, USA) for image acquisition. Cells were transfected with* ZFP91 *siRNA (ON-TARGET*plus* SMARTpool, Dharmacon) or negative control siRNA (ON-TARGETplus Nontargeting control Pool, Dharmacon) or left untreated. DharmaFECT Transfection Reagents 2 and 3 (Dharmacon) were used as transfection agents. The procedure was performed on logarithmically growing LNCaP and PC-3 cells according to manufacturer's recommendations with several modifications of the procedure tested. Viability of cells was determined by microscopic evaluation and trypan blue exclusion test.

### 2.4. Total RNA and Protein Extraction

Total RNA and protein were extracted by means of NucleoSpin RNA/Protein and NucleoSpin RNA Clean-Up XS (Macherey-Nagel Ltd., Oensingen, Switzerland). RNA concentration and purity were determined spectrophotometrically (NanoDrop, Thermo Scientific, Waltham, USA). For each sample 1 *μ*g of total RNA was reversely transcribed using MMLV reverse transcriptase kit (Novozym, Poznan, Poland) with Oligo dT (PE Biosystems, Warrington, UK) as primers. The reaction was performed at 42.8°C for 60 min (UNO II thermocycler, Biometra, Goettingen, Germany).

### 2.5. QPCR Analysis

**Figure 1:**
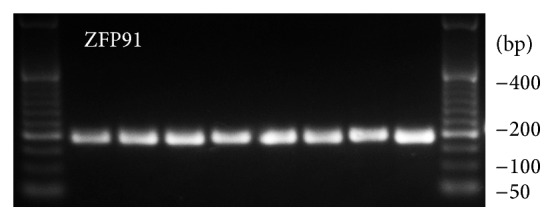
Ethidium bromide-stained 2.5% agarose gel showing random samples out of prostate cancer cDNA Array amplified with* ZFP91* primers. Note presence of reaction products with the expected size of 190 bp. As a DNA size standard O'RangeRuler 50 bp DNA Ladder (Fermentas) was used.

Analyses were performed as described earlier [2, 18, 19]. Briefly, primers were designed by Primer 3 software (Whitehead Institute for Biomedical Research, Cambridge, USA) and purchased from the Laboratory of DNA Sequencing and Oligonucleotide Synthesis (Institute of Biochemistry and Biophysics, Polish Academy of Sciences, Warsaw, Poland). Primers sequences are listed in Table 1. Real-time PCR was carried out in a LightCycler 2.0 thermocycler (Roche Diagnostics, Basel, Switzerland) with software version 4.05. SYBR Green detection system was used based on LightCycler FastStart DNA Master SYBR Green I mix (Roche). PCR reactions were carried out in 20 *μ*L mixtures, containing 4 *μ*L template cDNA, 0.2 *μ*M of each gene specific primer, and 3.5 mM of Mg2+ ions. The real-time PCR program included a 10 min denaturation step to activate the Taq DNA polymerase, followed by a three-step amplification program: denaturation at 95.0°C for 9 s, annealing at 58.0°C for 5 s, and extension at 72.0°C for 5 s. Specificity of the reaction products was routinely checked by determination of melting points (0.1°C/s transition rate) and random sample separation in a 2.5% ethidium bromide/agarose gel (Figure 1). PCR efficiency was assessed by a serial dilution method. Briefly, products of PCR reactions were separated in a 2.5% agarose gel and specific bands were extracted using a DNA gel extraction kit (Millipore, Billerica, USA). The amount of extracted DNA was estimated spectrophotometrically. Extracted DNA was diluted (10-fold serial dilutions) in order to generate a standard curve for efficiency calculation (LightCycler software version 4.05.).

### 2.6. Western Blot Analysis

For each sample, 20 *μ*g of protein was separated in a 4–20% gradient SDS-polyacrylamide electrophoretic gel and transferred onto a PVDF membrane. Transferred proteins were stained with Ponceau S. Membranes were incubated in a blocking buffer consisting of 5% nonfat dry milk in TBST for 1 h, followed by primary antibody incubation overnight at 4°C with rabbit anti-ZFP91 at 1 : 200 (sc-102172; Santa Cruz Biotechnology, Dallas, USA) or (to further validate the results) with rabbit anti-ZFP91 at 1 : 200 (ab30970; Abcam, Cambridge, UK) and with rabbit anti-*β*-Actin at 1 : 2000 (#5142; Cell Signaling Technology, Danvers, USA). Afterwards, membranes were thoroughly washed and incubated with an anti-rabbit HRP-linked antibody at 1 : 2000 (#7074; Cell Signaling Technology) for 1 h at room temperature. After washing, membranes were incubated with enhanced chemiluminescence (ECL Plus, Amersham, GE Healthcare) detection reagents (5 min, room temperature) and visualized on GelDoc-It Imaging System (UVP, Upland, USA) with use of VisionWorks LS Software. ECL DualVue Western Blotting Markers (GE Healthcare) served as a protein size standard. Band detection and quantification of band intensity were performed using TotalLab (Nonlinear dynamics, Newcastle upon Tyne, England).

### 2.7. Statistical Analysis

GraphPad Prism version 5.00 (GraphPad Software, San Diego, USA) was used to perform statistical analyses. Differences were considered significant at *p* < 0.05. For a more detailed description of particular experiment statistics, see description below each figure.

## 3. Results

### 3.1. *ZFP91 *mRNA Expression in Prostate Cancer Specimens and Its Correlation with Gleason Score

**Figure 2:**
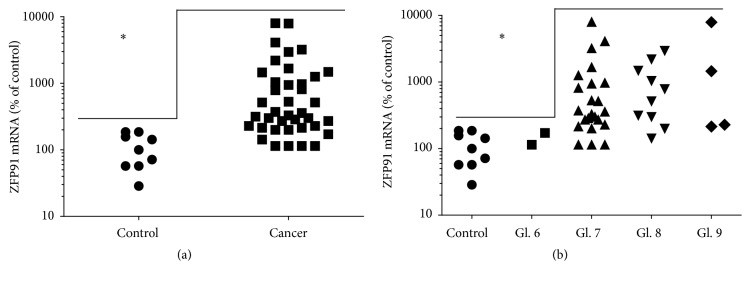
QPCR analysis of* ZFP91* gene expression in normal prostates (control *n* = 9) compared to prostate cancer specimens (Cancer *n* = 39) (a) and compared to prostate cancer specimens grouped according to Gleason score (Gl. 6 *n* = 2, Gl. 7 *n* = 23, Gl. 8 *n* = 10, and Gl. 9 *n* = 4) (b). Results are presented as a scatter plot and median expression in control group was assigned a value of 100. Statistical comparison by Mann-Whitney test (a) and by Kruskal-Wallis test followed by Dunn's test (b); ^*∗*^ *p* < 0.05.

Samples with cDNA from 48 patients with pathologically confirmed prostate cancer were examined. Samples came from tissue fragments evaluated microscopically as normal prostate or prostate cancer. Using QPCR method* ZFP91* expression was analyzed in above-mentioned samples finding its significant overexpression in prostate cancer (Figure 2(a)). In some cases the difference was over 10-fold. On the other hand, the range of* ZFP91* expression in cancer samples was very wide and specimens with similar expression as in control group were also present.

In subsequent analysis, samples taken from prostate cancer fragments were divided according to the Gleason score (Figure 2(b)). No significant differences in* ZFP91* expression were noted between tissues with Gleason scores 7, 8, and 9. Samples with Gleason score 6 prostate cancer had* ZFP91* expression similar to control group, that is, significantly lower. However, this interesting result requires further studies due to limited number of samples in Gleason score 6 group.

### 3.2. *ZFP91* siRNA Mediated Knockdown in LNCaP and PC-3 Prostate Cancer Cells

**Figure 3:**
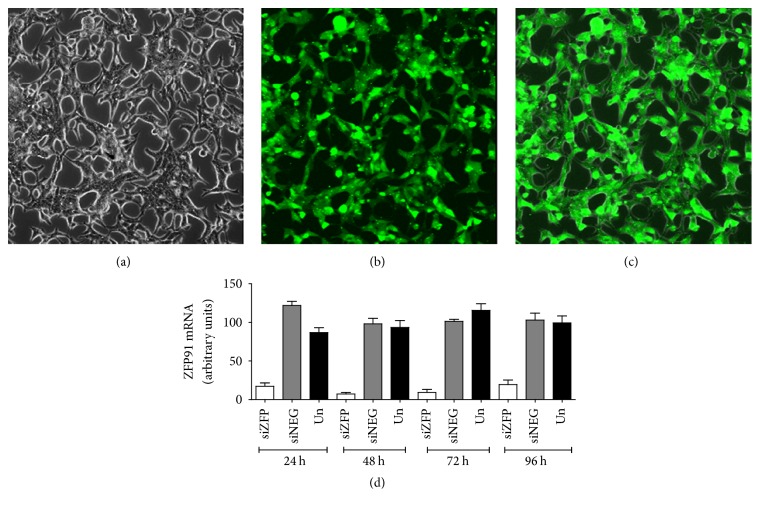
Transfection of LNCaP cell line with* ZFP91* siRNA. Top. cells transfected with siGLO Green Transfection Indicator showing good transfection efficiency. Representative cell images under bright field (a), fluorescence field with a FITC filter (b), and superimposed images (c). (d) QPCR analysis of* ZFP91* gene RNA interference. Cells transfected with* ZFP91* siRNA pool (siZFP), nontargeting siRNA control pool (siNEG), and untreated (Un).* ZFP91* mRNA levels at different time points were examined. Bars represent mean* ZFP91* expression ± SE; median value in untreated group was assigned a value of 100. Each experiment was performed in triplicate.

In order to optimize conditions for* ZFP91* gene knockdown in prostate cancer cells, LNCaP and PC-3 cell lines were firstly transfected with fluorescent indicator of transfection efficiency (siGLO Green Transfection Indicator, Dharmacon). After a series of experiments a protocol was established allowing successfully transfecting almost all cells in a culture (a representative image for LNCaP cells is shown in Figure 3). In a next step, using a pool of predesigned* ZFP91* siRNA (ON-TARGET*plus* SMARTpool, Dharmacon) transfection conditions were further optimized in order to achieve maximal* ZFP91* knockdown while maintaining unaffected cell viability.* ZFP91* mRNA downregulation to the levels between 5 and 20 percent of control groups was achieved in LNCaP cells (Figure 3). Similar results were obtained in PC-3 cells (data not shown). What is important, the silencing effect of RNA interference was stable over at least 4 days of cell culture (Figure 3).

### 3.3. Discrepancy between* ZFP91 *mRNA and Protein Levels

**Figure 4:**
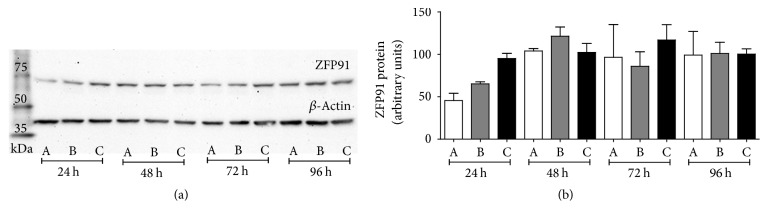
Representative experiment of ZFP91 protein immunoblotting in LNCaP cells transfected with* ZFP91* siRNA pool (A), nontargeting siRNA control pool (B), and untreated (C) at indicated time points. Bars represent protein expression relative to *β*-Actin levels. Median expression in untreated group was assigned a value of 100. Results are presented as means ± SE; each experiment was at least duplicated.

Protein levels of ZFP91 were examined in prostate cancer cells treated with* ZFP91* siRNA. Despite marked* ZFP91* mRNA downregulation no effect on the protein level was observed.  Figure 4 shows representative results of ZFP91 immunoblotting in LNCaP cells. Similar results were obtained for PC-3 cells (data not shown). What is important, this discrepancy between mRNA and protein levels was maintained over time of the cell culture. This finding was further validated by a set of experiments using, among others, different culture and transfection conditions and different ZFP91 primers and antibodies.

## 4. Discussion

The results of the present study show significantly elevated expression of* ZFP91* mRNA in prostate cancer samples compared with normal prostate, in some cases over 10-fold. These results are in accordance with an observation of Lee et al. of increased* ZFP91* mRNA staining in prostate cancer specimens using in situ hybridization method [6]. What is important, the hereby presented results come from a significant number of samples: 48, with full pathology reports available. As found in our previous work, similar* ZFP91* mRNA overexpression was not noted in prostate cancer cell lines, despite upregulated ZFP91 protein levels in these cells. This difference, although surprising, could be explained by genome differences between cancer cell lines and cancer specimens [20–23].

Analysis of* ZFP91* gene expression in prostate cancer samples subdivided according to Gleason score did not reveal significant differences between Gleason scores 7, 8, and 9. Lower* ZFP91* expression in samples with Gleason score 6 is an interesting result as it represents a low-risk prostate cancer and efforts are still being made to distinguish low- and high-risk disease [24]. However, the sample number in Gleason score 6 group is insufficient to draw any conclusions and this issue requires further study. Overall, no evidence suggests that observed* ZFP91* overexpression in prostate cancer is correlated with Gleason score or disease staging (data not shown). In this regard, it would be of interest to study potential differences between patients with high* ZFP91* expression in cancer samples and those with its expression unchanged. In the field of prostate cancer studies, genes whose expression is an independent risk factor of the disease course have been discovered [25]. Whether* ZFP91* gene expression could be such a factor remains to be answered. It is possible that it only accompanies other cellular processes (e.g., NF-*κ*B pathway activation) and as such does not represent an independent risk factor. Abundance and complexity of factors influencing NF-*κ*B pathway activation cause problems in selecting key compounds in the studied processes [26]. It would be important to test significance of the observed changes at the protein level, especially as marked discrepancies between mRNA and protein levels of* ZFP91* have been described. Marked overexpression of* ZFP91* in many samples of prostate cancer remains unclear and requires further investigation.

Studies on* ZFP91* gene's potential role in prostate cancer pathogenesis seem important not only due to its oncogenic properties. In recent years one of the thoroughly investigated molecular targets in prostate cancer therapy is factors involved in the NF-*κ*B pathway activation. Its role as a key regulator of immune processes is well documented; still an increasing line of evidence implies its influence on cancer initiation and propagation. With regard to prostate cancer biology, particularly underlined are multidimensional relations between NF-*κ*B pathway activation and activation of signaling pathways dependent on androgen receptor [27]. Interaction between androgen receptor and p52 factor (engaged in noncanonical NF-*κ*B pathway activation) promotes prostate cancer cell growth, protects from apoptotic cell death, and influences expression of androgen receptor dependent genes [28–31]. Taking this into consideration,* ZFP91* may potentially influence androgen receptor signaling in prostate cancer cells and therefore affect the biology of prostate cancer.

With regard to NF-*κ*B pathway function in prostate cancer cells, the influence of the canonical NF-*κ*B pathway activation agonists such as TNF and IL-1*β* is relatively well characterized. Less is known in this field regarding its noncanonical pathway. Functions of this additional and more specific NF-*κ*B activation pathway are not thoroughly studied; however available data suggest its involvement in prostate cancer biology [10, 27]. In vitro stimulation of cancer cells with TNF and LIGHT (peptide activating both canonical and alternative NF-*κ*B signaling pathway) stimulates* ZFP91* expression (in MCF-7 cells for TNF and HeLa cells for LIGHT) in a dose dependant manner [6, 8]. TNF influence on growth and function of prostate cancer cells in vitro has been well documented and, among other things, it is proven to induce apoptosis in LNCaP cells [32]. As for LIGHT, it enhances the proliferation rate of LNCaP cells [33]. Another agonist of both NF-*κ*B signaling pathways, RANKL, stimulates migration of PC-3 cells and expression of genes associated with metastatic potential in these cells [34]. ZFP91 protein as a factor specifically regulating NF-*κ*B noncanonical pathway activation [7, 8] may play an important role in above-mentioned relationships.

The present study is the first to our knowledge that directly tests the inhibition of* ZFP91* expression in prostate cancer cell lines using RNA interference. In the work of Unoki et al.* ZFP91* antisense oligonucleotides induced cell growth arrest and apoptosis in endometrial and colon cancer cell lines [4]. Lee and Jin et al. found that siRNA knockdown of* ZFP91* inhibited activity of HIF-1*α* and its dependent genes in colon cancer cell lines and induced apoptosis in breast cancer and stomach cancer cell lines [6, 9]. In our hands successful* ZFP91* mRNA knockdown was established in both LNCaP and PC-3 prostate cancer cell lines. On a protein level, however, no inhibitory effect was noted. Such phenomenon has been described in the literature and stems from only transient character of siRNA induced knockdown in cell culture [35, 36]. In cases of proteins with long turnover rate or proteins accumulated/stabilized in certain cell types siRNA mediated transfection does not result in phenotypic response. As found in our previous study, both LNCaP and PC-3 cell lines have elevated ZFP91 protein levels compared to normal prostate epithelial cells [2]. Taking this into consideration, together with a fact that ZFP91 protein knockdown was established in other cell lines, it may be hypothesized that in case of prostate cancer cells ZFP91 protein is accumulated or stabilized. Such posttranscriptional regulation of protein abundance has been described for other oncogenic proteins, for example, protein p53, where its accumulation involves enhanced translation of its mRNA and decreased proteolytic degradation [37]. It seems that in case of* ZFP91* expression no direct correlation exists between its mRNA and protein level, as described in our previous report and noted also in works of Lee and Jin et al. [2, 6]. Available data imply that* ZFP91* expression is subjected, at least partially, to a potent posttranscriptional regulatory mechanism. What is important, ZFP91 relative abundance in prostate cancer cells may play a role in this cancer biology.

A recent interesting study of Huang et al. presented effects of RNA-mediated knockdown of* ZFP91* pseudogene on biology of pancreatic cancer cells [38]. Such a silencing resulted in decreased pancreatic cancer cells proliferation rate, inhibition of their migratory ability, and a reversal, at least partial, of the epithelial to mesenchymal transition process in these cells. Long noncoding RNA (lncRNA) expressed by* ZFP91* pseudogene would be therefore another one in an emerging group of pseudogene lncRNAs involved in cellular development and cancer pathogenesis. Although no direct relation with* ZFP91* gene expression or function could be assigned to this, it is a relevant information as lncRNAs are capable of acting, for example, through generation of endogenous siRNAs and influencing mRNAs in the cell [39]. Whether results observed by Huang et al. are somewhat connected with interference in* ZFP91* expression requires further study.

In conclusion, recently discovered oncogenic properties of* ZFP91* should draw more attention to this subject and propel research regarding expression patterns and functions of this little-studied gene. The results presented in this study indicate that* ZFP91* is overexpressed in prostate cancer. A siRNA mediated knockdown of the* ZFP91* gene revealed a potential accumulation of the ZFP91 protein in prostate cancer cells. Taking into consideration known ZFP91 functions, this could play a role in NF-*κ*B and HIF-1*α* signaling in prostate cancer. In order to explore the significance of* ZFP91* in prostate cancer biology further studies are definitely needed.

## Figures and Tables

**Table 1:** Oligonucleotide sequences of sense (S) and antisense (A) primers are shown for ZFP91 zinc finger protein (ZFP91), two primer pairs, tubulin alpha 1b (TUBA1B), 5′-aminolevulinate synthase 1 (ALAS1), and *β*2-microglobulin (B2M).

cDNA	GenBank accession number	Primer	Primer sequence (5′-3′)	Position	PCR product size (bp)
ZFP91	NM_053023	S	TGTCCTTGCCCATCCTCGCTA	1128–1148	190
		A	ACTCTTGAAGGCCCGAGCAC	1298–1317	
		S	GAAACCCCAAAGCCACGGAG	892–911	227
		A	CCTTCCATCTCACAACGGACA	1098–1118	
TUBA1B	NM_006082	S	TGGAACCCACAGTCATTGATGA	430–451	135
		A	TGATCTCCTTGCCAATGGTGTA	543–564	
ALAS1	NM_000688	S	AGACATAACATCTACGTGCAA	2031–2051	167
		A	GAATGAGGCTTCAGTTCCA	2179–2197	
B2M	NM_004048	S	CAGCCCAAGATAGTTAAGTG	385–404	262
		A	CCCTCCTAGAGCTACCTGT	628–646	
